# Arsenic transport by zebrafish aquaglyceroporins

**DOI:** 10.1186/1471-2199-10-104

**Published:** 2009-11-25

**Authors:** Mohamad Hamdi, Marco A Sanchez, Lauren C Beene, Qianyong Liu, Scott M Landfear, Barry P Rosen, Zijuan Liu

**Affiliations:** 1 Dept. of Biological Sciences, Oakland University, Rochester, MI 48309, USA; 2 Dept. of Molecular Microbiology and Immunology, Oregon Health & Science University, Portland, OR, USA; 3 Oregon Hearing Research Center, Oregon Health & Science University, Portland, OR, USA; 4 College of Medicine, Florida International University, Miami, FL, USA

## Abstract

**Background:**

Arsenic is one of the most ubiquitous toxins and endangers the health of tens of millions of humans worldwide. It is a mainly a water-borne contaminant. Inorganic trivalent arsenic (As^III^) is one of the major species that exists environmentally. The transport of As^III ^has been studied in microbes, plants and mammals. Members of the aquaglyceroporin family have been shown to actively conduct As^III ^and its organic metabolite, monomethylarsenite (MAs^III^). However, the transport of As^III ^and MAs^III ^in in any fish species has not been characterized.

**Results:**

In this study, five members of the aquaglyceroporin family from zebrafish (*Danio rerio*) were cloned, and their ability to transport water, glycerol, and trivalent arsenicals (As^III ^and MAs^III^) and antimonite (Sb^III^) was investigated. Genes for at least seven aquaglyceroporins have been annotated in the zebrafish genome project. Here, five genes which are close homologues to human AQP3, AQP9 and AQP10 were cloned from a zebrafish cDNA preparation. These genes were named *aqp3, aqp3l, aqp9a, aqp9b *and *aqp10 *according to their similarities to the corresponding human AQPs. Expression of *aqp9a, aqp9b*, *aqp3, aqp3l *and *aqp10 *in multiple zebrafish organs were examined by RT-PCR. Our results demonstrated that these aquaglyceroporins exhibited different tissue expression. They are all detected in more than one tissue. The ability of these five aquaglyceroporins to transport water, glycerol and the metalloids arsenic and antimony was examined following expression in oocytes from *Xenopus leavis*. Each of these channels showed substantial glycerol transport at equivalent rates. These aquaglyceroporins also facilitate uptake of inorganic As^III^, MAs^III ^and Sb^III^. Arsenic accumulation in fish larvae and in different tissues from adult zebrafish was studied following short-term arsenic exposure. The results showed that liver is the major organ of arsenic accumulation; other tissues such as gill, eye, heart, intestine muscle and skin also exhibited significant ability to accumulate arsenic. The zebrafish larvae also accumulate considerable amounts of arsenic.

**Conclusion:**

This is the first molecular identification of fish arsenite transport systems and we propose that the extensive expression of the fish aquaglyceroporins and their ability to transport metalloids suggests that aquaglyceroporins are the major pathways for arsenic accumulation in a variety of zebrafish tissues. Uptake is one important step of arsenic metabolism. Our results will contribute to a new understanding of aquatic arsenic metabolism and will support the use of zebrafish as a new model system to study arsenic associated human diseases.

## Background

Arsenic is a well known environmental toxin and Group A human carcinogen. Humans are exposed to arsenic via a variety of other geochemical and anthropogenic sources, especially drinking water. The two major oxidation states of inorganic arsenic in water are trivalent arsenite (As^III^) and pentavalent arsenate (As^V^) [1]. Arsenate is usually reduced to arsenite in the cytosol of cells [2,3]. Usually, in well water and surface water, both As^III^, the more toxic form, and As^V^, the oxidized but less toxic form co-exist at variable ratios [4]. Since water is the major source of chronic exposure, most countries limit the upper level of arsenic allowed in drinking water. In the United States, the EPA lowered the maximal containment limit (MCL) from 50 ppb to 10 ppb http://www.epa.gov/EPA-WATER/2001/January/Day-22/w1668.htm. In Oakland County, Michigan (the location of Oakland University), wells contaminated with arsenic that leaches out of the Marshall Sandstone result in chronic arsenicosis in individuals dependent on those wells (front page of the Detroit Free Press, November 19, 1997; Flint Journal, Feb 14, 2008).

Aquaporins (AQPs) are integral membrane channel proteins that mediate the bidirectional downhill flux of water and selected small amphipathic molecules across cellular membranes [5]. Aquaglyceroporins are a subfamily of aquaporins that translocate larger molecules such as glycerol. Inherited mutation of AQPs is associated with multiple physiological disorders [5]. In total, twelve human aquaporins have been reported, and four of them, AQP3, AQP7, AQP9 and AQP10, are aquaglyceroporins. These AQPs play important roles in the tissues where they are expressed. For example, AQP7 is an adipose isoform that releases glycerol into the blood stream during lipolysis, while AQP9 is a liver isoform that uptake the glycerol [6,7]. The interplay of these two AQPs is critical for gluconeogenesis during starvation [8]. In addition to their physiological roles in nutrient transport, aquaglyceroporins have been shown to facilitate uptake of trivalent metalloids arsenite and antimonite because in solution they are trihydroxylated inorganic mimics of glycerol [9]. AQPs including *E. coli *GlpF [10], Yeast FPS1 [11], plant Nodulin26-like Intrinsic Proteins (NIPs) [12] and mammalian AQP3, AQP7 and AQP9 [13] were shown to transport arsenite. We have previously demonstrated mammalian AQP9 transports both inorganic As^III^, and its cellular methylation product, MAs^III ^[14]. Since AQP9 is a bi-directional channel, we have proposed that it mediates both influx of arsenite from blood into liver and efflux of MAs^III ^from liver to blood.

Fish, along with other aquatic organisms, are known to accumulate significant amounts of arsenic as well as other toxic metals such as mercury, copper, cadmium and nickel. For example, feral marine fish usually contain from 1 μg As/g dry mass to more than 10 μg/g dry mass [15]. In contrast, the reported levels for terrestrial animals, such as cattle or chickens, are only in the range of 7 - 30 ng As/g dry mass [16]. Although there are many studies concerned with general arsenic accumulation and speciation in various types of fish, there have been no mechanistic studies of arsenic detoxification in fish. Since zebrafish is the most studied fish model, in this study, we examined the capacity of zebrafish aquaglyceroporins for metalloid uptake. By querying the zebrafish annotated genome database with the sequence of the human AQP9, seven members of aquaglyceroporins were indentified. These Aqps share high sequence similarity with the corresponding human AQPs. Only one, termed *aqp3*, has been characterized to date [17]. In this study, we demonstrate that five zebrafish aquaglyceroporins are functionally expressed in *Xenopus *oocytes and transport water, glycerol and the metalloids As^III^, MAs^III ^and trivalent antimonite (Sb^III^). These Aqps are differentially expressed in a variety of zebrafish organs. Thus, the extensive expression of Aqps may account for the accumulation of arsenic in different tissues. The identification of arsenic metabolism pathways in fish is important to understand the arsenic transformation mechanisms in aquatic organisms and to evaluate the safety of fish as a food.

## Methods

### Protein sequence alignment

**Figure 1:**
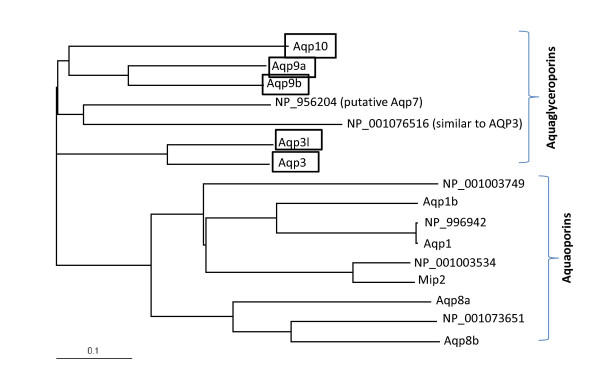
**Phylogenetic tree of the zebrafish aquaporin and aquaglyceroporin family**. Zebrafish sequences were obtained by blasting the protein database with human AQP9 and AQP1. Proteins with different sequences were chosen to be aligned.

**Figure 2:**
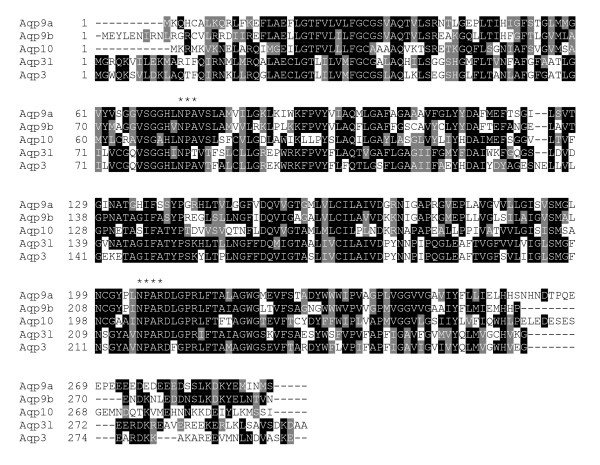
**Sequence alignment of five zebrafish aquaglyceroporins**. Black background represents the identical residues while grey background represents the similar residues.

The protein sequences were obtained from NCBI. The alignment was done using ClustalW http://www.ebi.ac.uk/Tools/clustalw2/index.html. The phylogenetic tree was constructed using TreeViewX http://darwin.zoology.gla.ac.uk/~rpage/treeviewx/ (Fig. 1). To perform a detailed protein sequences alignment, the sequences were first allinged with ClustalW, and then performed by Boxshade server http://www.ch.embnet.org/software/BOX_form.html. Sequences in black background indicate identical residues while in grey background means similar residues (Fig. 2).

### RNA isolation, cDNA synthesis and RT-PCR

To generate a full-length cRNAs, the total RNA was extracted from isolated adult organs as well as the 72-hours post fertilization (hpf) embryos (Invetrogen, Trizol) and reverse-transcribed to single-stranded cDNA using a commercial kit (Superscript III, Invitrogen Co.) according to the manufacturer's instructions. RT-PCR was performed using specific primers (Table 1. The primers were designed according the published sequences and synthesized commercially (IDT DNA). The optimum annealing temperature of each gene was determined by gradient PCR. Where the average melting temperature (T_m_) of the forward and reverse primers for each gene was used for setting the annealing temperatures for the gradient PCR. The annealing temperatures ranged from (Average T_m_-5°C) to the elongation temperature (72°C).

**Table 1:** Primers used for cloning of zebrafish Aqps.

Genes	Primers (5' TO 3')
*aqp9a *(882 bp)	Forward: *GAT GAA GCA GCA CTG CGC GCT C*
	Reverse: *GCT AGC TCA TGT TGA TCA TCT C*
	
*aqp9b *(873 bp)	Forward: *ATG GAG TAT CTC GAG AAC ATC*
	Reverse: *CTA GTT GAC GGT GTT CAG CTC*
	
*aqp3l *(888 bp)	Forward: *ATG GGA AGA CAG AAG GTA ATC C*
	Reverse: *TTA TGC TGC ATC TTT ATC AG*
	
*aqp3 *(897 bp)	Forward: *CAG ATC TAT GGG TTG GCA GAA AAG*
	Reverse: *CAG ATC TTC ATT CCT TGC TGG CGA CGT C*
	
*aqp10 *(879 bp)	Forward: *ATG AAG AGG ATG AAG GTG AAA AAT G*
	Reverse: *TTA AAT TGA AGA CAT TTT AAG GTA TAT CTC*

Some RT-PCR products in the studies of tissue expression were partially sequenced by using forward primers in Table 2. Specifically, RT-PCR products of *aqp9a *from intestine and kidney, *aqp9b *from eyes, *aqp3 *from eye, *aqp3l *from eye and skin, *aqp10 *from intestine, kidney and liver were verified by partial sequencing.

### Identification and cloning of aquaglyceroporin genes from zebrafish cDNA

The sequence of human AQP9 was used to identify zebrafish Aqps from the sequences in the NCBI zebrafish database. Seven different zebrafish aquaglyceroporin genes were annotated. Unique primers were designed to clone five closest human AQP3, AQP9 and AQP10 homologues. The following PCR conditions were employed: preheat 94°C for 2 min; denature 94°C 1 min; anneal 45°C for 1 min; elongate 72°C for 1 min; repeat 30 cycles. A second round of PCR was done if the amount of product from the first round was too low. The PCR products were ligated into vector plasmid pGEMT-easy (Promega Co.) or PL2-5 [18] and their sequences were verified by DNA sequencing (Beckman CEQ2000).

### Expression of zebrafish aquaglyceroporins in Xenopus oocytes

The capped RNA from five zebrafish Aqps were transcribed *in vitro *using mMessage mMachine T7 ultra kit (Ambion Co.), as described previously [18]. Stage V-VI *Xenopus *oocytes were isolated and treated by 0.2% collagenase A (Roche) for 2 hours. The defoliated oocytes were injected with 10 ng of different cRNAs. They were then incubated in ND96 buffer (96 mM NaCl, 2 mM KCl, 1 mM MgCl_2_, 1.8 mM CaCl_2_, 5 mM Hepes, pH7.5) for 3 days at 16°C and used for uptake assays [19].

### Water osmolarity measurement

P_f _was determined by placing oocytes in 50% ND96 buffer and swelling was recorded using a microscopy (Nikon LABOPHOT-2) and camera system (SPOT PURSUIT, Diagnostic Instruments Inc.) at 10 sec intervals for 1 min. The images were processed by ImageJ software http://rsbweb.nih.gov/ij/. The water permeation coefficient (P_f_) was calculated as reported previously [20].

### Transport assays

*In vivo *metalloid uptake assays were performed as previously described [18]. In brief, oocytes were incubated in either 0.5 mM ^3^H-glycerol, 1 mM sodium arsenite (Sigma), 1 mM sodium monomethylarsenite (MAs^III^) (a gift from William Cullen at the University of British Columbia, Canada) or 1 mM potassium sodium tartrate antimonite (Sigma), which all dissolved in ND96 buffer. After incubation at room temperature for 30 min, the oocytes were collected and washed in ND96 buffer three times. The oocytes were either treated with 10% SDS for scintillation counting or with 70% nitric acid at 70°C for metalloid quantification. One oocyte was used each time and at least seven replicates were used to calculate the standard error. The amount of arsenic and antimony was determined by inductivity coupled plasma mass spectrometry (ICP-MS, ELAN 9000, PerkinElmer, Norwalk, CT). Zebrafish tissues or *Xenopus *oocytes were completely digested with 0.1 ml of 70% (vol/vol) HNO_3 _for two hours. The samples were then diluted to 2 mL final volume with HPLC grade water for metalloid assays.

### Arsenic treatment for zebrafish larvae and adults

The zebrafish (AB line) larvae were treated with 5 ppm (46 μM) of sodium arsenite (Sigma) in 20 mL water from 2 to 6 days post fertilization (dpf). Adult zebrafish were treated with arsenic by placing 5 ppm sodium arsenite in I L water continuously for 96 hr. The water that containing arsenic was changed every day with fresh arsenite to avoid oxidation to arsenate. Following arsenic exposure, the fish were transferred to arsenic-free fresh water for one hour to remove nonspecifically bound arsenic. The zebrafish were euthanized with 0.2 mM tricaine (MCA222, Sigma). Each data point represents three samples, and each sample contains ten larvae, which were pooled and homogenized. Various adult organs, including as brain, heart, muscle, liver, eye, skin, intestine and gill, were isolated from six fish and analyzed for arsenic accumulation. These samples were weighed, and total arsenic was determined by inductively coupled plasma mass spectrometry (ICP-MS), as described above.

### Statistical Analysis

Quantitative results are shown as means ± standard deviations. The results were subjected to statistical analysis using single-factor analysis of variance (ANOVA). *P *values < 0.05 were considered significant.

## Results

### Nomenclature of zebrafish aquaglyceroporins

Three human aquaglyceroporins, AQP3, AQP7 and AQP9, have been shown to facilitate arsenite influx with different efficiencies [21]. AQP9 is the major liver isoform and shows the highest rate of arsenite conduction *in vitro *[22]. In a query of the zebrafish database with human AQP9, seven distinct zebrafish aquaglyceroporin homologues have been located. Zebrafish aquaporin water channel homologues were identified by querying with human AQP1. Fig. 1 shows a phylogenetic tree of the orthodox aquaporins and aquaglyceroporins. The sequences of five aquaglyceroporins that have been studied are aligned in Fig, 2.

Among these seven aquaglyceroporins, one has been characterized as an amphipathic channel and was named *aqp3 *[17]. The other six are uncharacterized open reading frames (ORF). In this study, four out of these six genes that encodes the aquaglyceroporin were cloned and studied. Through discussion with the Zebrafish Nomenclature Committee, and in accordance with the committee's principles [23], these genes have been named *aqp9a, aqp9b, aqp3l *and *aqp10*, according to the similarity of their gene products to the human gene products (boxed in Fig. 1). Unfortunately, the other two genes were not successfully obtained by RT-PCR and their function remains unknown (NCBI accession number NP_956204 and NP_001076516). These four zebrafish Aqps, along with Aqp3, share high sequence identities with the human AQPs (44%-65%) and are most highly conserved at the two NPA/NPAR motifs (Indicated in Fig. 2). The NCBI access number and the names of these genes, along with their protein sequence identities and similarities are listed in Table 2.

**Table 2:** Overall identities and similarities of zebrafish aquaglyceroporins with human homologues.

Zebrafish Aqps (NCBI access number)	Zebrafish Aqps (Current names)	Human AQPs	Identities %	Similarities %
NP_001028268	*aqp9a*	AQP9	52	77
XP_698730	*aqp9b*	AQP9	49	72
NP_998633	*aqp3*	AQP3	63	78
XP_696449.1	*aqp3l*	AQP3	65	81
NP_001002349	*aqp10*	AQP10	44	68

### Tissue specific Aqp expression

**Figure 3:**
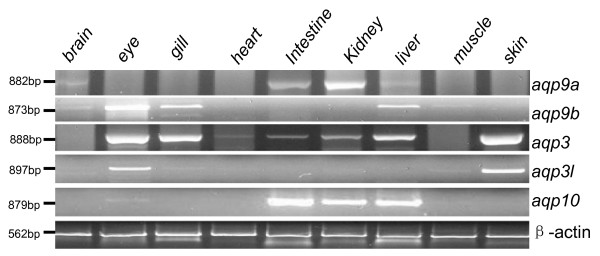
**Expression of mRNA of zebrafish aquaglyceroporins**.The expression of mRNA of *aqp9a*, *aqp9b*, *aqp3, aqp3l*, and *aqp10 *in isolated zebrafish tissues. Tissues were isolated from adult zebrafish, homogenized, and total cDNA was obtained. A zebrafish β-actin gene was used to be a positive control.

Expression of *aqp *genes in adult zebrafish tissues was examined by RT-PCR. The aquaglyceroporins exhibited different patterns of expression. The *aqp3 *was expressed in multiple tissues, including eye, gill, heart, intestine, kidney, liver and skin (Fig. 3). These results are consistent with data from in-situ hybridization in zebrafish larvae that showed expression of Aqp3 in gill and skin (Bernard and Thisse, ZFIN direct submission). The expression of Aqp3 in embryonic eye was also previously reported (zfin direct submission, http://www.zfin.org)[24,25]. Using RT-PCR, it has been found that Aqp3 can be detected in more adult tissues in addition to these three organs (gill, skin and eye) that are detected by in-situ hybridization (zfin direct submission). The first possibility is that RT-PCR is a more sensitive approach to study mRNA transcription. Second, the Aqp3 may be developmentally regulated. The close Aqp3 homologue, Aqp3l, shows different tissue expression when compared with Aqp3. Aqp3l was mainly detected in eye and skin (Fig. 3). Aqp10 was expressed in intestine, kidney and liver (Fig. 3). Aqp9a was detected in brain, intestine, kidney and liver while Aqp9b was detected in brain, eye, gill and liver (Fig. 3). The differential expression of the individual *aqp *gene results in all examined tissues expressing at least one type of aquaglyceroporin.

Similar to other animals, zebrafish aquaglyceroporins have a differential but extensive tissue expression pattern. The Aqp3 is unique member that can be detected in most tissues except for brain and muscle. The expression of Aqp3 has been studied in many other fish species. It has been found that in different fish, Aqp3 has a different expressional pattern. For example, in fresh water and sea water tilapia, Aqp3 is present in brain, pituitary, kidney, spleen, intestine, skin, eye and gill [26]. While in silver sea bream, Aqp3 was detected in gill, kidney, liver, brain, heart, and spleen [27]. It appears that the gill and skin are sites where Aqp3 can be found in many types of fish.

### Functional expression of zebrafish aquaglyceroporins in Xenopus oocytes

**Figure 4:**
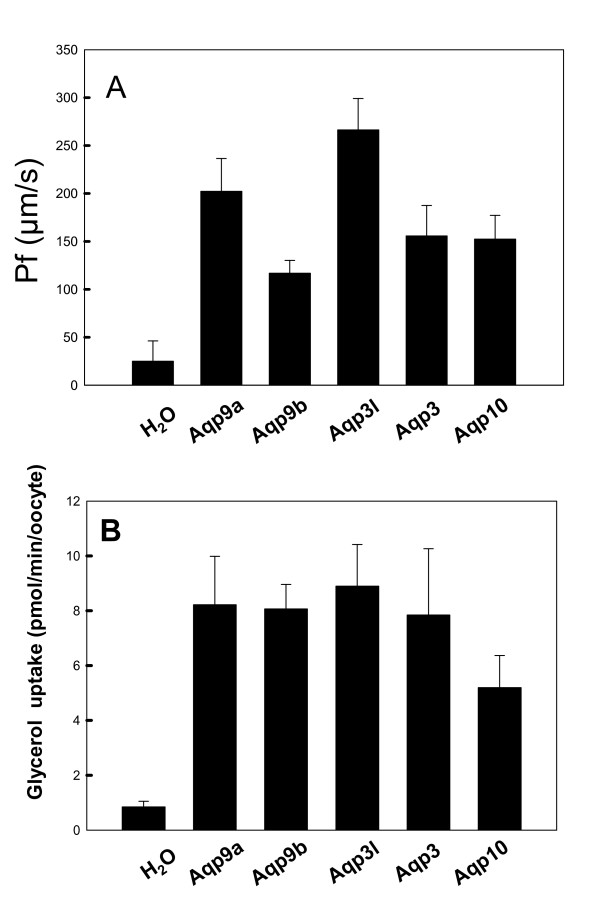
**Water permeation and glycerol transport by zebrafish AQP-expression *Xenopus oocytes***. **A**. Water osmolarity (P_f_) is determined in oocytes that have expressed zebrafish aquaglyceroporin genes. (n = 5 oocytes were used. *P *< 0.01 is calculated from water control and Aqp injected samples). **B**. Glycerol transport is determined by applying 0.5 mM [^3^H] glycerol for 30 min. The standard deviation was calculated using Sigma Plot 10 (n = 7 oocytes were used from same frog, *P *< 0.01 is calculated from water control and Aqp injected samples).

Five aquaglyceroporin genes, *aqp9a*, *aqp9b*, *aqp3*, *aqp3l*, and *aqp10*, were cloned using a cDNA library of whole adult zebrafish (a gift from Jeffery Yoder, North Carolina State University). The 5' capped RNA made *in vitro *was microinjected into *Xenopus *oocytes. Oocytes expressing any of the five transporters conduct water or glycerol uptake efficiently compared with control (Fig. 4). Water and glycerol are two typical substrates for aquaglyceroporins. The results clearly demonstrated that all these five aquaglyceroporins are expressed in *Xenopus *oocytes and function as aquaglyceroporins.

### Metalloid transport

**Figure 5:**
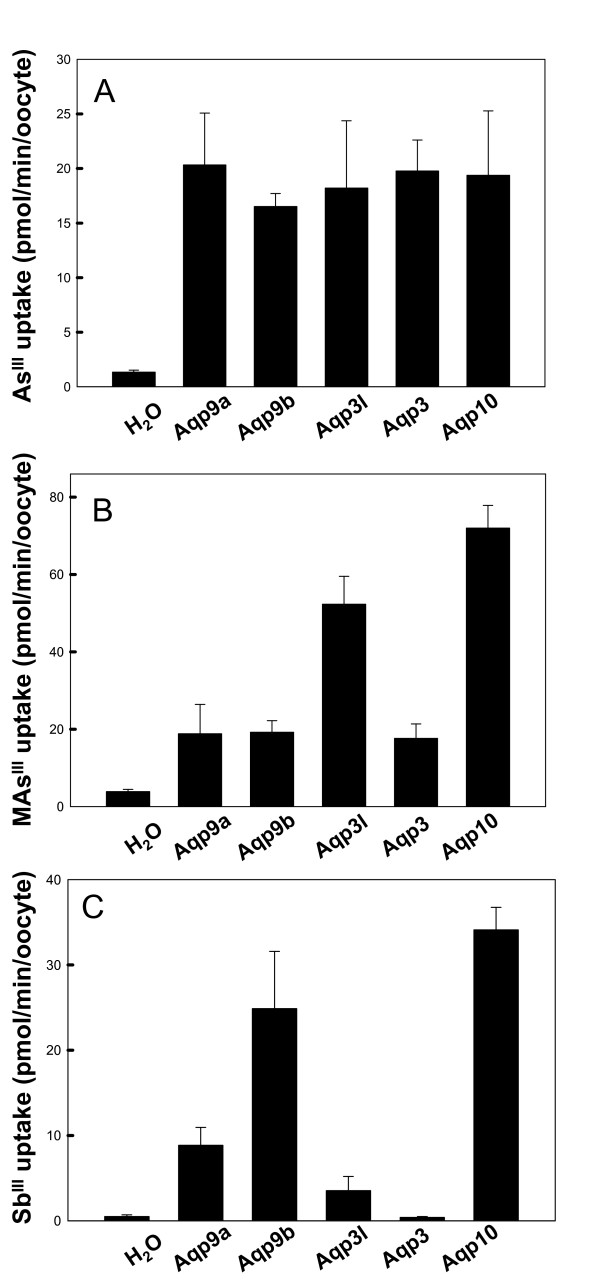
**Uptake of As^III^, MAs^III ^and Sb^III ^by zebrafish AQP-expression *Xenopus oocytes***. Various metalloids were added at 1 mM final concentration to oocytes that were injected by cRNA. Oocytes were incubated for 30 min and digested for metalloid quantification. The standard deviation was calculated from three replicates and plotted using Sigma Plot 10 (n = 7 oocytes were used, *P *< 0.01 calculated from water control and Aqp injected samples). **A**. Uptake of As^III^. **B**. Uptake of MAs^III^. **C**. Uptake of Sb^III^.

Metalloid transport by the five zebrafish aquaglyceroporins was examined. Each Aqp facilitated uptake of either As^III ^or MAs^III ^(Fig. 5A and Fig. 5B). Overall, three Aqps facilitate organic MAs^III ^at higher rates than inorganic As^III^, which is consistent with our previous observation with mammalian AQP9 [14]. Oocytes expressing *aqp3l *and *aqp10 *exhibit 4 and 6-fold increases in the rate of MAs^III ^uptake compared with As^III ^uptake, respectively. (Fig. 5A &5B). It appears that rate of As^III ^transport is comparable to glycerol transport, and both are relative lower than the transport of MAs^III^. Transport of trivalent antimony (Sb^III^) was also examined. In the periodic table, the metalloid antimony is in the same column as arsenic, and it shares similar chemical properties. Antimony is also a toxic metalloid that exists in aquatic surroundings. Oocytes expressing *aqp9a*, *aqp9b *or *aqp10 *exhibited Sb^III ^permeation, but oocytes expressing *aqp3I *did not (Fig. 5C). Oocytes expressing *aqp9b *or *aqp10 *exhibited higher rates of Sb^III ^transport than for As^III ^(The transport rate of As^III ^and Sb^III ^were analyzed by t-test, and *P *< 0,01 is calculated from the indicated pairs).

### Effect of arsenic exposure on arsenic accumulation in zebrafish tissues

**Figure 6:**
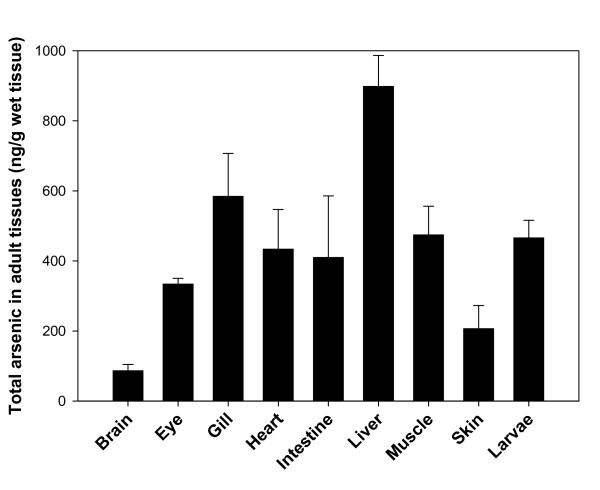
**Effect of short-term arsenic exposure on accumulation of arsenic in zebrafish**. After arsenic treatment, organs from adult fish were isolated (n = 6) and homogenized. Totally 10 zebrafish larvae were pooled as one sample (n = 4). Standard deviation is determined by Sigma plot 10.

Arsenic is primarily a water-borne pollutant. Inorganic arsenic, including trivalent arsenite and pentavalent arsenate, are often found in natural waters [4]. Fish accumulate arsenic through mouth and gills. Since arsenite is more toxic than arsenate, arsenic accumulation was assayed in zebrafish larvae and adult tissues following arsenite exposure. Zebrafish larvae accumulated considerable amounts of arsenic (Fig. 6), which is consistent with our observation that larvae express *aqp *genes (data not shown). Adult fish tissues accumulated arsenic in different amounts. As is the case with humans, arsenic accumulates most highly in liver. Arsenic exposed fish had enlarged livers (data not shown), which has been observed also in humans [28]. Arsenic accumulated in gill, muscle, heart, eye, liver, intestine and skin. Brain is the only organ that exhibited limited ability to accumulate arsenic (Fig. 6). It is shown that zebrafish larvae accumulated as much arsenic as adult muscle, demonstrating that these channels which can take up arsenic are expressed early in development.

## Discussion

It has been previously demonstrated that metalloids, including arsenic, antimony, boron and silicon are transported by microbial, mammalian and plant aquaglyceroporins [5,29]. Since arsenic is a major water-borne contaminant, it is reasonable to speculate that fish uptake and accumulate this toxic element. Fish are already known for their high tolerance and accumulation of toxic metals and metalloids [30,31]. These elements are taken up via gills or consumed in the diet. However, the specific molecular mechanisms involved in arsenic accumulation and metabolism are not known. As uptake is the first step in arsenic metabolism, the first question to ask is what transporters are responsible for the observed high levels of arsenic accumulation. Since aquaglyceroporins are known to transport both inorganic arsenite [13] and organic MAs^III ^[14] in other species, it is logical to investigate the function of aquaglyceroporins in zebrafish. Aquaporins play an important role in water balance in all organisms including aquatic organisms [17]. Aquaglyceroporins may have additional functions in glycerol metabolism and cold tolerance, as this is the case in other organisms [32]. In this study, we cloned five zebrafish aquaglyceroporins, *aqp9a, aqp9b, aqp3, aqp3l *and *aqp10*. Their function in water, glycerol and metalloid As^III^, MAs^III ^and Sb^III ^transport was studied in *Xenopus laevis *oocytes. All five are efficient water/glycerol transporters. In addition, each of these aquaglyceroporins actively conducts metalloid arsenic and antimony transport with one exception that Aqp3 does not transport antimonite. These properties indicate that *aqp9a, aqp9b, aqp3, aqp3l *and *aqp10 *have a unique importance in normal physiological function as well as in toxin metabolism.

Metalloid transport by human aquaglyceroporins has been well characterized. The liver isoform, AQP9, has a high rate of transport of both As^III ^and MAs^III ^[18]. Intracellular arsenic undergoes a series of oxidative methylations and reductions in many mammals, including humans. These methylations and oxidations are catalyzed by the enzyme AS3MT, an arsenic methyl-transferase [33], and result in the production of the organic mono- and di-methylated arsenic species MAs^III ^(monomethylarsonous acid), MAs^V ^(monomethylarsonic acid), DMAs^III ^(dimethylarsonous acid) and DMAs^V ^(dimethylarsonic acid) [34]. MAs^III ^is believed to be an early intermediate in the process of arsenic methylation in liver. It has been proposed that AQP9 facilitates the efflux of MAs^III ^which is produced in the liver and transported to the blood and eventually urine [14].

Similar to humans, multiple zebrafish aquaglyceroporins transport both environmental As^III ^and intracellularly generated MAs^III^. This observation provides a basis to position zebrafish as an excellent model organizm to study arsenic-associated diseases. Additionally, the zebrafish model has a number of practical advantages. The zebrafish is a vertebrate with rapid embryonic development and organ differentiation, and its entire genome has been sequenced. As such, the zebrafish fills the gap between microorganisms and mammals. Furthermore, it has a short generation time that allows for statistical analysis of large groups. Many zebrafish genes exhibit high sequence similarity to their human homologues. For example, the zebrafish aquaglyceroporin genes examined in this study are close homologues to aquaglyceroporin genes found in humans. Additionally, the zebrafish fish is native to streams of the Ganges region in eastern India, Pakistan, Bangladesh and Nepal, where the concentrations of arsenic are of the highest in the world. The zebrafish commonly inhabits streams, canals, ditches, ponds, and slow-moving to stagnant water bodies, including rice fields, which are often contaminated with arsenic. It would not be surprising if the zebrafish has adapted to this environmental exposure by expressing arsenic detoxification genes. In this study, we demonstrated that five zebrafish aquaglyceroporins facilitate the uptake of both As^III ^and MAs^III^. This is similar to the results found with human AQP9. These five Aqps were shown to have different tissue localizations. This may partially explain why multiple adult organs accumulate large amounts of arsenic. While there is a lack of autopsy data to show the differential arsenic accumulation in human tissues, studies with rats show that after a single oral dose of arsenite, arsenic accumulates primarily in the liver and followed by the kidney [35].

With more mechanistic studies of arsenic metabolism in zebrafish, it will be possible to clarify how environmental arsenic is detoxified by aquatic animals. Zebrafish is a promising model to simulate this process in other fish, including multiple commercial fish species such as tilapia, salmon and trout. By examining arsenic metabolism in zebrafish, we will discover valuable information to evaluate the safety of fish that is consumed as a food. With further studies, it may be possible to produce genetically modified zebrafish for use as a bio-monitor to evaluate environmental arsenic contamination.

## Conclusion

Our studies demonstrate that five zebrafish aquaglyceroporins actively conduct water and glycerol, which is consistent with functions in osmoregulation. In addition, these five aquaglyceroporins conduct the metalloids arsenic and antimony, which is consistent with the observation that larvae and selected adult organs exhibit significant arsenic accumulation. Our results indicate that zebrafish can become a promising animal model to study arsenic-associated cancers and non-cancer-related diseases.

## Abbreviations

As^III^: arsenite; As^V^: arsenate; MAs^III^: monomethylarsenite; DMA^III^: dimethylarsenite; DMA^V^: dimethylarsenate; TMAO: trimethylarsine oxide; TMA^III^: trimethylarsine; Aqp/AQP: aquaporin/aquaglyceroporin; ICP-MS: inductively coupled plasma mass spectrometer; dpf: days post fertilization; hpf: hours post fertilization; ORF: open reading frame.

## Authors' contributions

MH carried out the arsenic accumulation study in different zebrafish tissues and the RT-PCR, as well as sequencing of RT-PCR products. MAS performed cRNA microinjection of *Xenopus *oocyte. Lauren Beene participated in the cDNA preparation and manuscript editing. QL participated in the cDNA isolation and discussed the project. SML providing funding support and communicate in the manuscript. BPR: participated in the experimental design, provided funding support and helped to draft the manuscript. ZL designed the overall experiments, carried out the gene cloning and transport assay, performed the sequence alignment and drafted the manuscript. All authors read and approved the final manuscript.
